# Discrepancies between estimated and geometrically derived mechanical power in invasively ventilated patients – insights from a randomized clinical trial

**DOI:** 10.1186/s40635-026-00956-8

**Published:** 2026-08-18

**Authors:** Simon Corrado Serafini, Jan Willem H. J. Geerts, Ary Serpa Neto, Stephanie S. List, Stephan von Düring, Edda Tschernko, Philipp Bühler, Guido Mazzinari, Frederique Paulus, Marcus J. Schultz, Laura A. Buiteman–Kruizinga

**Affiliations:** 1 Department of Intensive Care, Amsterdam University Medical Centers, ‘Location AMC’, Amsterdam, the Netherlands; 2 Department of Surgical Sciences and Integrated Diagnostics, Università Di Genova, Genoa, Italy; 3 Department of Intensive Care, Reinier de Graaf Hospital, Reinier de Graafweg 5, Delft, the Netherlands; 4 Australian and New Zealand Intensive Care–Research Centre (ANZIC–RC), Monash University, Melbourne, Australia; 5 Department of Intensive Care, Austin Hospital, Melbourne, Australia; 6 Department of Critical Care, University of Melbourne, Melbourne, Australia; 7 Department of Critical Care Medicine, Hospital Israelita Albert Einstein, São Paulo, Brazil; 8 Department of Intensive Care, Dijklander Hospital ‘Location Hoorn’, Hoorn, the Netherlands; 9 Division of Emergency Medicine, Department of Acute Care Medicine, Geneva University Hospitals, Geneva, Switzerland; 10 Department of Anaesthesiology, Pharmacology, Intensive Care and Emergency Medicine, Geneva University Hospitals, Geneva, Switzerland; 11 Department of Anesthesia, General Intensive Care and Pain Management, Medical University Wien, Vienna, Austria; 12 Center of Intensive Care Medicine, Cantonal Hospital Winterthur, Winterthur, Switzerland; 13 Department of Intensive Care Medicine, University Hospital Zurich, Zurich, Switzerland; 14 Department of Anaesthesiology and Critical Care, La Fe University and Polytechnic Hospital, Valencia, Spain; 15 ACHIEVE, Centre of Applied Research, Faculty of Health, Amsterdam University of Applied Sciences, Amsterdam, The Netherlands; 16 Mahidol–Oxford Tropical Medicine Research Unit (MORU), Mahidol University, Bangkok, Thailand; 17 Nuffield Department of Medicine, University of Oxford, Oxford, UK

**Keywords:** Intensive care, Critical care, Mechanical ventilation, Ventilation, Mechanical power of ventilation, Mechanical power, MP, Geometric MP

## Abstract

**Background:**

Mechanical power (MP) quantifies the energy transferred from the ventilator to the respiratory system and is increasingly linked to outcomes in invasively ventilated critically ill patients. Limiting MP may support lung-protective ventilation by integrating key determinants of ventilator-induced lung injury. While geometric MP, derived from pressure–volume loops, is considered the reference standard, it is not readily available at the bedside. Therefore, simplified equations are commonly used, although their agreement remains uncertain. This study evaluates the agreement of previously proposed equations to estimate inspiratory MP compared with a geometric reference.

**Results:**

494 paired values of geometric and estimated MP were collected, in 38 passively ventilated patients during closed-loop ventilation and during conventional ventilation. The median geometric MP was 17.0 [12.3–21.5] J/min. Correlation between geometric and estimated MP ranged from strong to very strong. Agreement between geometric and estimated MP was good, though bias and limits of agreement varied across the different equations. ROC analysis showed excellent discrimination for most equations across all tested cutoffs, with discrimination gradually declining at higher MP levels.

**Conclusion:**

In passively ventilated critically ill patients, estimated MP showed strong to very strong correlations with the geometric reference. ROC analysis confirmed excellent discrimination, but Bland–Altman analysis revealed considerable variability, suggesting potential limitations in using these equations interchangeably in clinical or research settings.

**Study registration:**

INTELLiPOWER was registered at clinicaltrials.gov (study identifier NCT04827927).

**Supplementary Information:**

The online version contains supplementary material available at 10.1186/s40635-026-00956-8.

## Introduction

Mechanical power of ventilation (MP) represents the energy transferred from the ventilator to the respiratory system over time. Several equations have been proposed to estimate MP in studies in invasively ventilated critically ill patients [1–8]. Previous studies have demonstrated associations between MP and outcomes [9–13], while more recent work suggests that the elastic components of MP may drive much of this association [14]. As a result, limiting MP and its components has been proposed as a promising lung-protective ventilation strategy, as it integrates multiple ventilator variables implicated in ventilator-induced lung injury (VILI).

Despite its increasing use, there is ongoing debate regarding the definition of MP. Clinical definitions generally include both resistive and elastic components, thereby reflecting total ventilator-delivered energy transfer and allowing bedside assessment. From a physiological perspective, however, the energy received, stored, and returned by the respiratory system may be more relevant to VILI, although this cannot be directly measured in routine clinical practice. Consequently, given that directly measurable bedside alternatives are lacking, correct estimation and careful interpretation of currently available MP measurements are essential to assess their physiological relevance and clinical implications.

Geometric MP is derived from ventilator waveforms by integrating the area under the inspiratory limb of the pressure–volume loop to measure inspiratory tidal energy, which is multiplied by the respiratory rate. Although no physiological gold standard for MP exists, geometric MP as a waveform-derived inspiratory energy represents a comprehensive bedside-available method of ventilator-derived energy transfer, and has therefore been used previously as a reference method for inspiratory estimates [4, 15–18]. However, geometric MP is also not available at the bedside. Instead, clinicians and researchers typically rely on mathematical equations ranging from simple bedside formulas to more complex models. The extent to which these equations approximate waveform-derived inspiratory energy remains uncertain.

Using ventilator waveform data from a recent randomized clinical trial comparing closed–loop with conventional ventilation in critically ill patients [19], the aim of this study was to evaluate the agreement between inspiratory MP calculated from ten previously proposed equations and a reference based on geometric MP. We also assessed their discrimination in identifying excessive MP using Receiver Operating Characteristic (ROC) curves. We hypothesized that previously proposed MP equations would demonstrate strong agreement with geometric MP.

## Methods

### Design, study oversight and ethics

This is a preplanned secondary analysis of ‘The Effect of Closed–loop versus Conventional Ventilation on Mechanical Power’ (INTELLiPOWER) study, an investigator–initiated, international, multicenter, randomized crossover clinical trial that compared two modes of invasive ventilation [19]. In INTELLiPOWER, patients were randomly assigned to start with a 3–hour period of closed–loop using INTELLiVENT–adaptive support ventilation or conventional pressure controlled or pressure support ventilation, after which the alternate ventilation mode was selected. The trial was conducted in three intensive care units (ICUs) in the Netherlands (Reinier de Graaf Hospital in Delft, the Dijklander hospital in Hoorn, the Amsterdam University Medical Centers in Amsterdam), and one ICU in Switzerland (University Hospital Zürich in Zürich). The study protocol was approved by the institutional review board of the AMC (2020_317#B2021122) and the Cantonal Ethics Commission Zurich (Swissethics, 2023–D0012). Written informed consent was obtained from legal representatives of all patients prior to inclusion and randomization. INTELLiPOWER was registered at clinicaltrials.gov (study identifier NCT04827927). The primary analysis showed a reduction in MP with closed-loop ventilation compared to conventional ventilation in passively ventilated patients, but no difference was seen in the overall cohort [20].

### Patients

Patients were eligible for participation in INTELLiPOWER if: (1) aged ≥ 18 years; (2) expected to need invasive ventilation for > 24 h; (3) having the ability to randomize as soon as possible, but always < 48 h after start of invasive ventilation in the ICU; and (4) ventilated with a ventilator that was able to provide the closed–loop ventilation mode of interest. Patients with a tracheostomy, any contraindication for use of the closed–loop ventilation mode, or a body mass index (BMI) exceeding 40 kg/m^2^ were excluded.

Patients were eligible for participation in this pre–planned secondary analysis if waveform data was recorded, as this was needed for calculating the geometric MP. Waveform data were collected in all participating centers. We additionally excluded patients who were actively breathing at any time point during the study.

### Data collected

Demographic data and baseline characteristics were collected for each patient. Ventilator settings and parameters were manually recorded every 30 min, including tidal volume (V_T_), respiratory rate (RR), peak pressure (Ppeak), maximum airway pressure (Pmax), plateau pressure (Pplat), inspiratory pressure (Pinsp), positive end–expiratory pressure (PEEP), inspiration (Tinsp) and expiration (Texp) time, inspiration rise time (Tslope) and flow (F).

Ventilation waveform data was collected with a sampling rate of 50 Hz and stored on a mass storage device (MemoryBox, Hamilton Medical AG). Data collection included the date and time of each breath and all waveform characteristics, i.e. flow, pressure and volume.

### Calculations

Driving pressure (ΔP), respiratory system compliance (C_RS_) and necessary parameters to calculate MP with some of the equations were calculated as follows, using the manually collected data:

i $$\rm \Delta P (cmH_{2}O)\hspace{0.17em}=\hspace{0.17em}Pplat (cmH_{2}O) - total\,PEEP(cmH_{2}O)$$

ii $$\rm C_{RS} (mL/cmH_{2}O)\hspace{0.17em}=\hspace{0.17em}V_{T} (mL) / \Delta P (cmH_{2}O)$$

V_T_ was normalized to predicted body weight (PBW), using the following equation for PBW:

iii $$\rm PBW\hspace{0.17em}=\hspace{0.17em}50.0\hspace{0.17em}+\hspace{0.17em}0.9 * (body\,height\,in\,cm-152.4)\,in\,males$$

iv $$\rm PBW=45.5+0.9 * (body\,height\,in\,cm-152.4)\,in\,females$$

### Geometric MP

Geometric MP, a waveform-based estimate of inspiratory energy transfer, was used as an operational reference because no gold standard for inspiratory MP exists. It reflects ventilator-delivered rather than patient-absorbed or dissipated energy. Geometric MP was calculated from ventilator waveform data as described before [1]. At each 30–minute time point, the first three consecutive breaths were manually selected to reconstruct the dynamic pressure–volume (P–V) loop. Tidal energy was measured by numerically integrating the area under the inspiratory limb of the P–V loop [17]. This energy was then multiplied by the corresponding RR to obtain MP:

v $$\rm Geometric\,MP\hspace{0.17em}=\hspace{0.17em}tidal\,energy*RR\,(J/min)$$

MP values from the three selected breaths were averaged to yield a single value for each time point.

### MP equations tested in the analysis

The identified equations range from simple bedside approximations to more comprehensive models for inspiratory MP, differing in their intended purpose, definitions, and assumptions regarding respiratory system mechanics, including resistance, compliance, flow profile, pressure rise time, and nonlinear pressure–volume behavior (eTable 1). The equations were as follows:

1 $$\rm MP (J/min)\hspace{0.17em}=\hspace{0.17em}0.098 \cdot VT \cdot RR \cdot (Ppeak - 1/2\cdot \Delta P)$$

2 $$\rm MP (J/min)\hspace{0.17em}=\hspace{0.17em}0.098 \cdot RR \cdot \{VT \cdot (PEEP\hspace{0.17em}+\hspace{0.17em}\Delta\,Pinsp)\}$$

3 $$\rm MP (J/min)\hspace{0.17em}=\hspace{0.17em}0.098 \cdot VT\hspace{0.17em}\cdot \hspace{0.17em}\Delta PRS \cdot \hspace{0.17em}RR$$

4 $$\rm MP (J/min)\hspace{0.17em}=\hspace{0.17em}0.098 \cdot RR \cdot VT \cdot Pplat$$

5 $$\rm MPdyn (J/min)\hspace{0.17em}=\hspace{0.17em}0.098 \cdot VT \cdot (Ppeak-(\Delta Pdyn) / 2) \cdot RR$$

6 $$\mathrm{M}\mathrm{P} (\mathrm{J}/\mathrm{m}\mathrm{i}\mathrm{n}) = \frac{\mathrm{V}\mathrm{E} \cdot (\mathrm{P}\mathrm{e}\mathrm{a}\mathrm{k} \mathrm{P}\mathrm{r}\mathrm{e}\mathrm{s}\mathrm{s}\mathrm{u}\mathrm{r}\mathrm{e} + \mathrm{P}\mathrm{E}\mathrm{E}\mathrm{P} +\frac{\mathrm{F}}{6})}{20}$$

7 $$\begin{aligned}&\mathrm{M}\mathrm{P} (\mathrm{J}/\mathrm{m}\mathrm{i}\mathrm{n})\\&=0.098\cdot \mathrm{R}\mathrm{R}\cdot {\mathrm{V}}_{\mathrm{T}}\cdot \left(\mathrm{P}\mathrm{E}\mathrm{E}\mathrm{P}+\Delta {\mathrm{P}}_{\mathrm{i}\mathrm{n}\mathrm{s}\mathrm{p}}\cdot \left(1-{\mathrm{e}}^{\frac{-{\mathrm{T}}_{\mathrm{i}\mathrm{n}\mathrm{s}\mathrm{p}}}{R\cdot C}}\right)\right)\end{aligned}$$

8 $$\begin{aligned}&\rm MP(J/\min ) \\&= 0.098 \cdot RR \cdot \\&\left\{ {\Delta VT2 \cdot \left[ {1/2 \cdot ELrs + RR \cdot \frac{(1 + I:E)}{{60*I:E}} \cdot Raw} \right]+ \Delta VT \cdot PEEP} \right\}\end{aligned}$$

9 $$\begin{aligned}& \rm MP (J/min)\\&=0.098 \cdot RR \cdot \{VT \cdot [PEEP+\Delta Pinsp]-0.15 \cdot \Delta Pinsp2+{T}_{slope}/ R\}\end{aligned}$$

10 $$\begin{aligned}&\mathrm{M}\mathrm{P} \left(\frac{\mathrm{J}}{\mathrm{m}\mathrm{i}\mathrm{n}}\right)\\&=0.098\cdot RR\cdot {\{V}_{T}\cdot \left(PEEP+\Delta {P}_{insp}\right)-{\Delta {P}_{insp}}^{2} \\&\cdot C\cdot \left(0.5- \frac{R\cdot C}{{T}_{slope}}+\left(\frac{R\cdot C}{{T}_{slope}}\right)2 \cdot \left(1-{e}^{\frac{{-T}_{slope}}{R\cdot C}}\right)\right)\}\end{aligned}$$

The identified equations range from simple bedside approximations to more comprehensive models for inspiratory MP, differing in their intended purpose, definitions, and assumptions regarding respiratory system mechanics, including resistance, compliance, flow profile, pressure rise time, and nonlinear pressure–volume behaviour (eTable 1).

### Outcomes

The primary outcome was the geometric MP; secondary outcomes were MP estimates from proposed equations.

### Statistical analysis

The number of available patients served as the sample size. Data are presented as medians with interquartile ranges (IQR) for continuous variables, and numbers with percentages for categorical variables. Effects are reported with a 95%–confidence interval (95%–CI). Correlations and AUROC values across a range of MP cutoffs are visualized in line plots, while Bland–Altman analyses are presented as plots showing bias and limits of agreement. For the latter, the mean difference between the MP values from each equation and the geometric MP was plotted against their average, allowing visualization and to assess systematic bias and determine limits of agreement (mean difference ± 1.96 standard deviations).

We performed a complete case analysis, meaning there were no missing values in every case. MP values were obtained at 30–minute intervals during the 6.5–hour study period, resulting in 13 sampling time points per patient. To compare MP calculated using the proposed equations with the geometric MP reference, we first conducted a mixed–effect generalized linear model with Gaussian distribution to account for the repeated measurements. Time of measurement was included as a continuous variable, MP equations as fixed effect and patients and ventilation mode as random effects. We also compared differences in absolute MP between closed–loop and conventional ventilation.

We first assessed the correlation between each equation and the geometric MP using Pearson’s correlation coefficient. Pearson’s correlation coefficients were interpreted according to conventional thresholds: values of 0.90 or higher indicating very strong correlation, 0.70 to 0.89 strong, 0.50 to 0.69 moderate, 0.30 to 0.49 weak, and below 0.30 negligible correlation.

To evaluate agreement and identify systematic bias we performed Bland–Altman analysis using geometric MP as the reference. For this, the mean difference between the MP values from each equation and the geometric MP was plotted against their average, allowing visualization and to assess systematic bias and determine limits of agreement (mean difference ± 1.96 standard deviations).

To explore performance across previously proposed MP cutoffs [3, 5, 11, 12, 21], we used a range of threshold values from 10 to 22 J/min and calculated the area under the ROC (AUROC) to assess each equation’s ability to identify patients exceeding the chosen geometric MP threshold. An AUROC of 0.90–1.00 indicated excellent discrimination, 0.80–0.89 good discrimination, 0.70–0.79 fair discrimination, 0.60–0.69 poor discrimination, and 0.50–0.59 failure or no discrimination. Optimal cutoff values were determined by maximizing the Youden index (sensitivity plus specificity minus 1). Sensitivity, specificity, positive predictive value, and negative predictive value were reported for these cutoffs.

We performed a sensitivity analysis limited to data from conventional ventilation, excluding measurements obtained during closed-loop ventilation, repeating the correlation analysis, Bland–Altman analyses and AUROC calculations.

Data were analyzed using R 4.4.2 (R Foundation, Vienna, Austria). A two–sided *P* value < 0.05 was considered statistically significant.

## Results

### Patients

**Fig. 1:**
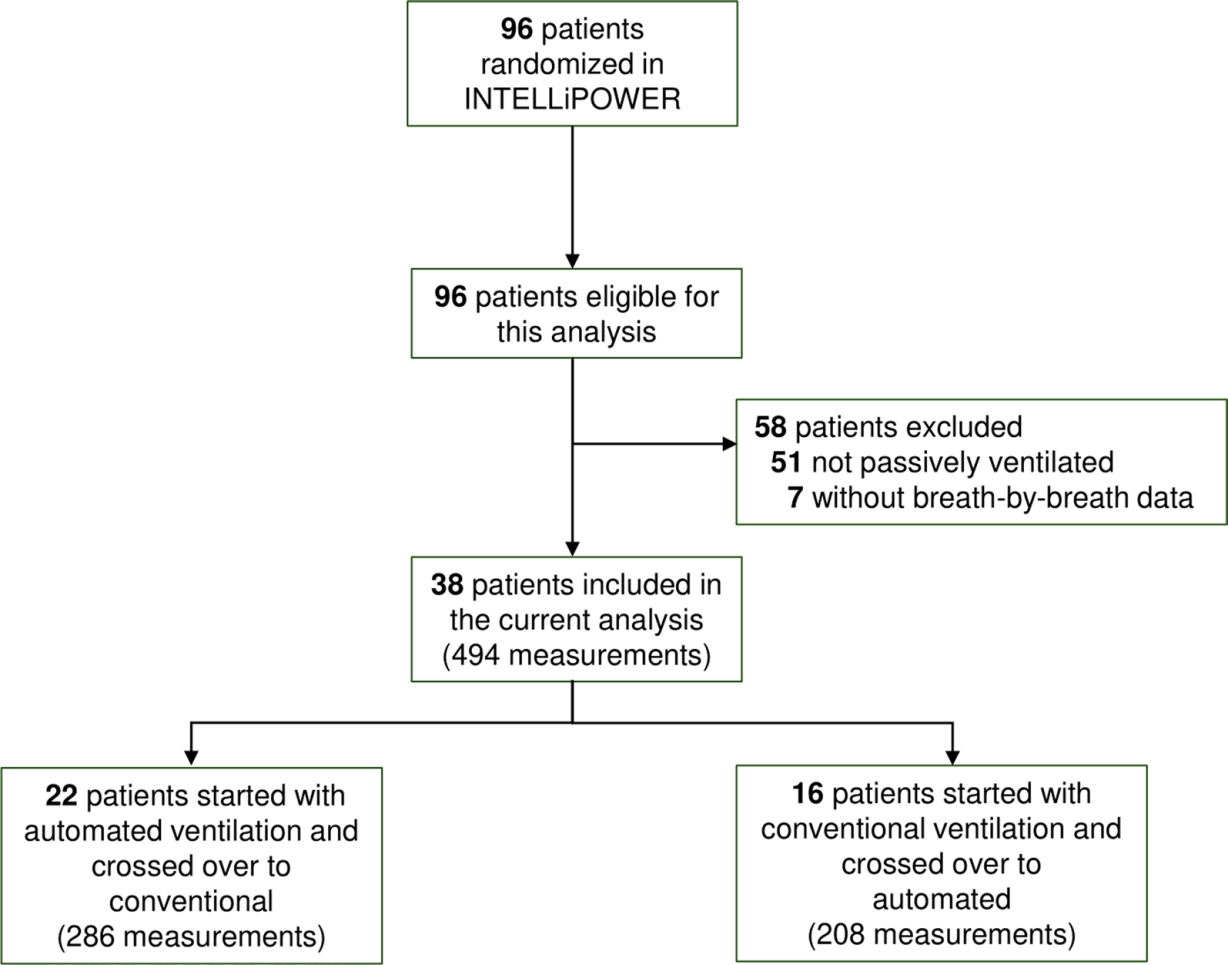
Flowchart. Flowchart of the patients included in the analysis

Of the 96 patients enrolled in INTELLiPOWER, 38 patients were included in the preplanned secondary analysis (Fig. 1). The main reason for exclusion was the presence of spontaneous breathing activity. Most patients were male, aged over 60 years, admitted for a medical reason, and receiving invasive ventilation due to respiratory failure (Table 1). The majority received ventilation with V_T_ < 8 ml/kg PBW and PEEP < 12 cm H_2_O, accompanied with a low ΔP at baseline (eTable 2). In total, 494 paired values of geometric and estimated MP were collected, with a median of 13 [13] pairs per patient; 6 [6] during closed–loop ventilation, and 6 [6] during conventional ventilation.

**Table 1:** Baseline characteristics

	**N = 38**
Age, year	66 [59–72]
Sex, female	5 (13)
Height, cm	175 [169–183]
Weight, kg	82 [75–90]
PBW, kg	71 [64–78]
BMI, kg/m^2^	27 [23–29]
APACHE IV score	75 [57–91]
Reason for ICU admission	
medical	33 (87)
elective surgery	1 (2)
emergency surgery	4 (11)
Comorbidities	
neurological	3 (34)
cardiovascular disease	21 (55)
COPD	3 (8)
Reason for mechanical ventilation	
acute respiratory failure	27 (71)
depressed consciousness	3 (8)
cardiac arrest	5 (13)
postoperative ventilation	2 (5)
other	5 (13)

Data are expressed as median with [IQR] or numbers n/N (proportions) where appropriate. Four patients had multiple reasons for mechanical ventilation

APACHE IV score, Acute Physiologic Assessment and Chronic Health Evaluation IV Score; BMI, Body Mass Index; COPD, Chronic Obstructive Pulmonary Disease; IQR, Interquartile range; PBW, Predicted body weight

### Geometric MP

Using the geometric method, the median MP in the entire cohort was 17.0 [12.3–21.5] J/min (eTable 3). MP was lower during closed–loop than conventional ventilation (15.0 [12.0–21.3] vs 18.6 [13.7–21.8] J/min, and this reduction was consistent across all comparisons (Table 2). MP estimates varied between the ten equations tested (eFigure 1).

**Table 2:** Differences in MP between closed–loop and conventional ventilation

Equations	Closed-loop ventilation (N = 22)	Conventional ventilation (N = 16)	Difference (%)	Mean difference (95% CI)	p value
Geometric method	15.0 [12.0–21.3]	18.6 [13.7–21.8]	21.08	ref	ref
Equation 1	14.7 [11.1–20.9]	16.9 [11.7–21.2]	2.65	–1.12 (–1.64 to –0.61)	< 0.001
Equation 2	18.3 [14.2–25.3]	21.1 [15.4–25.6]	14.49	2.95 (1.94 to 3.96)	< 0.001
Equation 3	7.7 [6.0–10.5]	9.0 [6.3–11.3]	15.55	−8.34 (−9.36 to −7.31)	< 0.001
Equation 4	15.9 [12.3–19.3]	17.4 [12.6–22.1]	9.00	−0.31 (−1.34 to 0.72)	0.55
Equation 5	13.3 [10.0–17.3]	15.4 [10.9–19.4]	14.18	−2.66 (−3.68 to −1.65)	< 0.001
Equation 6	17.4 [13.3–22.2]	19.3 [13.3–23.5]	10.64	1.51 (0.49 to 2.52)	0.003
Equation 7	17.3 [13.2–22.0]	19.9 [14.7–24.6]	13.71	1.75 (0.73 to 2.78)	0.001
Equation 8	14.7 [11.1–18.4]	16.7 [11.3–20.3]	12.55	−1.35 (−2.38 to −0.32)	0.01
Equation 9	18.0 [14.0–25.1]	21.0 [15.0–25.3]	15.19	2.61 (1.60 to 3.61)	< 0.001
Equation 10	17.0 [14.0–25.1]	20.1 [14.1–24.4]	10.89	2.50 (1.47 to 3.53)	< 0.001

Data, inherent to mechanical power, are expressed as median with [IQR]s in Joules/min. Difference in percentage is calculated as (MP closed-loop ventilation-MP conventional)/((MP closed-loop ventilation + MP conventional)/2)*100

CI; confidence interval, Eq.; Equation, Ref.; Reference

### Correlations between MP estimates and the geometric MP

**Fig. 2:**
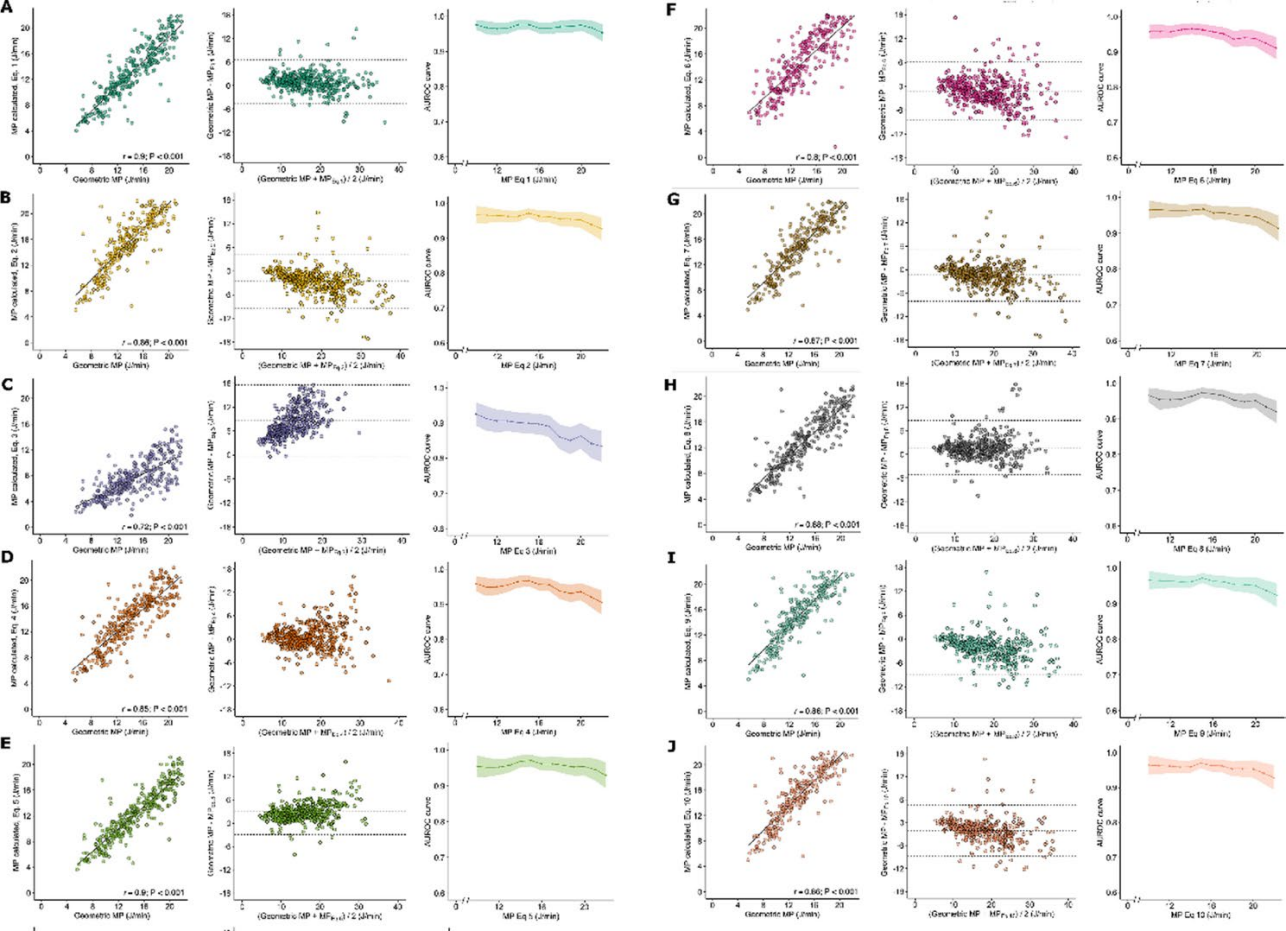
Correlation plots, Bland–Altman plots and ROC curves from all proposed equations, with the geometric method as the reference. Panels **A **to **J **represent Eqs. 1, 2, 3, 4, 5, 6, 7, 8, 9, and 10 , and are described in the Methods section. First and fourth column: correlation plots showing correlation between the geometric MP on the x-axis and the calculated MP for each equation on the y-axis. Each dot represents an individual patient measurement. Correlation was assessed using Pearson’s method and results are shown at the bottom right of the plot. Second and fifth column: Bland–Altman plots comparing geometric MP with each MP equation. The X-axis represents the mean values of MP, while the Y-axis shows the difference between means. The middle line shows the mean difference (bias) and the upper and lower line the limits of agreement. Third and sixth column: line plots showing AUROC values on the y-axis for each MP equation across thresholds ranging from 10 to 22 J/min, on the x-axis. Shaded areas represent 95% confidence interval

Correlations between estimated and geometric MP were strong across the equations, with Eqs. 1 and 5 demonstrating particularly high agreement (Fig. 2).

### Bland–altman analysis

Equations 1 and 4 exhibited minimal systematic bias, with values below 1, although the limits of agreement were wide. Equations 6, 7 and 8 showed higher bias, remaining below 2, but their limits of agreement were substantial. Equations 2, 5, 9 and 10 showed more bias, between 2 and 3, also with wide limits of agreement. Equation 3 demonstrated the poorest agreement (Fig. 2, Table 3).

**Table 3:** Differences between geometric MP and all equations

equations	mean of differences (bias)	standard Deviation	standard Error	within Subject Variance	between Subjects Variance	95% LOA (lower limit)	95% LOA (upper limit)
Equation 1	0.97	2.83	0.10	8.01	40.47	−4.58	6.52
Equation 2	−2.53	3.49	0.12	12.18	45.69	−9.37	4.31
Equation 3	8.71	4.55	0.16	20.73	20.87	−0.21	17.6
Equation 4	0.71	3.60	0.13	12.99	34.24	−6.35	7.78
Equation 5	2.99	3.04	0.11	9.23	32.36	−2.96	8.95
Equation 6	−1.18	3.74	0.13	13.96	41.50	−8.51	6.14
Equation 7	−1.40	3.42	0.12	11.72	42.83	−8.11	5.31
Equation 8	1.74	3.52	0.12	12.39	34.84	−5.16	8.64
Equation 9	−2.18	3.41	0.12	11.63	43.39	−8.87	4.50
Equation 10	−2.09	3.41	0.12	11.63	43.69	−8.77	4.60

Eq.; equation, LOA; limits of agreement

### ROC analyses

ROC curves showed excellent discrimination for all equations across cutoffs, except for Eq. 3. Performance generally declined with increasing MP (Fig. 2, eTables 4 to 16).

### Sensitivity analysis

The findings remained unchanged when only measurements from conventional ventilation were included. (eTables 4b to 16b, 17 & 18b).

## Discussion

In this analysis, we compared estimated MP calculated from ten previously proposed MP equations with geometric MP as a ventilator-derived operational reference. The main findings can be summarized as follows: 1) all equations showed a strong to very strong correlation with geometric MP; 2) overall, most equations demonstrated low systematic bias but wide limits of agreement in Bland–Altman analysis, indicating substantial variability; and 3) ROC analysis demonstrated excellent discrimination across equations, with performance declining at higher MP values.

There is no universal definition of MP, since it can be conceptualized from different perspectives, i.e. the clinical perspective, describing the energy delivered per unit time based on airway pressure and flow and directly measurable at the bedside, and the physiological perspective, relating to the energy absorbed, stored, and dissipated within the respiratory system, which cannot be directly quantified in clinical practice. Our analysis focused on the clinical perspective and comprehensively evaluated the agreement of all previously proposed MP equations by directly comparing them with the geometric method. Prior studies examined only a subset of equations [4, 15, 16, 18, 22], compared a new equation [4] to four others and geometric MP, while another [15] assessed two equations in patients with ARDS on different ventilation modes. Across modes, equations correlated with geometric MP [22], with two equations slightly underestimating MP but remaining within an acceptable range [18]. In artificial settings, several equations exhibited considerable bias compared with the geometric method [16]. Our analysis expands on these studies by providing a comparison of all existing approaches from the ventilator perspective.

The proposed equations capture different parts of the pressure–volume relationship, representing distinct physical quantities despite sharing the same units. In particular, inspiratory power, total-cycle dissipative power, and simplified algebraic approximations derived from the equation of motion are not mathematically or physiologically identical; some formulas should therefore be interpreted as simplified or surrogate estimates rather than full measures. From a physiological perspective, only dissipative energy, represented by the area within the full pressure–volume loop, reflects net energy transfer to the respiratory system, whereas elastic energy stored during inspiration is returned during expiration [23]. Previous work [1, 24–27] suggesting a relationship between energy load and lung injury has focused on carefully partitioned dissipative power, including separation of airway and chest wall components, an approach not feasible in routine clinical practice. This study did not address mechanistic validity or determine which formulation best reflects VILI biology; instead, it evaluates agreement between commonly used definitions of inspiratory MP and a waveform-based reference. The observed differences reflect divergence between mathematical formulations rather than validation of a specific mechanism.

In current practice, limiting MP has emerged as a key target in lung–protective ventilation, underscoring the need for accurate and reliable assessment of MP, particularly in patients at risk of VILI. The highest agreement was observed for equations designed to approximate waveform-derived inspiratory energy transfer, likely reflecting similarities in their underlying mathematical formulations. However, in practice, these formulas are often used interchangeably, leaving clinicians uncertain which approach to apply. Our study highlights this variability and lack of standardization. Notably, we found that simple equations provide good agreement with the geometric reference and perform at least as well as more complex formulas that rely on variables that are difficult to measure accurately at the bedside. Importantly, a universally accepted cutoff for “excessive” MP remains undefined. Although several thresholds have been proposed [3, 5, 11, 12, 21, 28], our findings show that discrepancies between estimation methods increase at higher MP levels, precisely where the risk of lung injury is greatest.

Our findings demonstrate that, although estimated MP values correlate well with the geometric method, systematic bias suggests that different MP equations cannot be used interchangeably. This limits the immediate use of MP as a target variable for ventilation strategies or guideline development and undermines comparability between studies using different estimation methods. The ICU community will need to agree on whether to adopt a single standardized approach or define one based on specific MP cutoffs. Simpler equations using routinely available ventilator variables (Eq. 1 and Eq. 4) showed good agreement with the geometric method, despite being originally designed for volume-controlled ventilation, and may offer a feasible interim solution where geometric MP is not available, depending on the intended application, MP cutoffs, and the typical MP range of the target population. However, integrating geometric MP calculation directly into ventilator software would be more appropriate and convenient, eliminating the need for surrogate equations and improving accuracy and reproducibility. In current practice, ventilators may already display MP values using different calculation methods, which can lead to inconsistencies.

This analysis has several strengths. The original study was a multicenter international randomized clinical trial examining the effects of a ventilation strategy on MP, enhancing data reliability. Waveform recordings were used to obtain geometric MP as an operational reference. The study was conducted in academic and non-academic hospitals, and included patients with varying MP levels, increasing generalizability. A sophisticated analysis plan was applied, extending beyond simple correlation analyses to include Bland–Altman and ROC analyses, thereby comprehensively assessing correlation, agreement and systematic bias. All analyses were predefined and consistently applied, underscoring methodological rigor. The cohort was sufficiently large with complete data, minimizing the risk of bias from missing information.

This analysis has limitations. We evaluated a short early phase of ventilation; results may differ later, and it is unclear whether agreement between geometric and estimated MP is independent of ventilation duration. Only passively ventilated patients were included, improving precision but limiting generalizability, although appropriate given the lack of validated equations for spontaneous breathing. The sample size precluded subgroup analyses. Data from closed-loop and conventional ventilation were combined without affecting results, and all equations were applied in pressure-controlled ventilation, reflecting common practice [20, 29]. This is a methodological comparison of formulas; while providing a comprehensive assessment of performance, it did not evaluate a recently developed equation for ventilation intensity [10] or assessed dissipative energy. Differences could arise from simplifying assumptions (e.g., linear pressure–volume relationships) that are violated in clinical conditions. These findings indicate that mechanical power formulations are not interchangeable but represent structurally different constructs. While no single preferred equation is identified, the results highlight lack of standardization and support harmonization of definition and clinical implementation rather than further formula development.

## Conclusion

In this cohort of passively ventilated critically ill patients, estimated MP correlated well with geometric MP and showed good agreement, but substantial variability across equations suggests caution when using these equations interchangeably, particularly at higher MP values. From a practical standpoint, the simpler equations may offer a feasible interim solution where geometric MP is not available .

## Supplementary Information

Supplementary material 1.

## Data Availability

The dataset used in this analysis that support the findings of this study is available from the authors upon reasonable request. A statistical analysis plan is requested and a datasharing agreement needs to be signed beforehand.

## Abbreviations

ΔP: Driving pressure
ΔPdyn: Dynamic driving pressure
ΔPinsp: Inspiratory pressure above PEEP
ΔPRS: Driving pressure of the respiratory system
ΔVT: Tidal volume
ΔVT2: Square of tidal volume
C: Compliance
e: Euler’s number, approximately 2.718
ELrs: Elastance of the respiratory system
IE: Inspiratory to expiratory ratio
MP: Mechanical power
MPdyn: Dynamic mechanical power
Ppeak: Peak airway pressure
Pplat: Plateau pressure
PEEP: Positive end-expiratory pressure
R: Resistance
Raw: Airway resistance
RR: Respiratory rate
Tinsp: Inspiratory time
Tslope: Inspiratory pressure rise time
VE: Expiratory minute volume
V_T_: Tidal volume
