# Supplementary material for: Discrepancies between estimated and geometrically derived mechanical power in invasively ventilated patients – insights from a randomized clinical trial
Source: Intensive Care Med Exp. 2026 Aug 18;14:107. doi: 10.1186/s40635-026-00956-8 (PMC13486459; doi:10.1186/s40635-026-00956-8)
Supplement: Supplementary file 1 [file 40635_2026_956_MOESM1_ESM.docx]

SUPPLEMENT TO:

**Discrepancies between Estimated and Geometrically Derived Mechanical Power in Invasively Ventilated Patients – insights from a randomized clinical trial**

Simon Corrado Serafini[+], Jan Willem H.J. Geerts[+], Ary Serpa Neto, Stephanie S. List, Stephan von Düring, Marcus J. Schultz, Edda Tschernko, Philipp Karl Bühler, Guido Mazzinari, Frederique Paulus, Laura A. Buiteman–Kruizinga, for the INTELLiPOWER^†^ collaborative investigators

+Authors contributed equally to the work

**Correspondence:**

Laura Buiteman–Kruizinga, RN PhD

Department of Intensive Care

Reinier de Graaf Hospital

Reinier de Graafweg 5, Delft

The Netherlands

Email: L.Kruizinga@rdgg.nl

**Table of contents	page**

eTable 1	Assumptions and purpose of proposed MP equations	3

eTable 2	Pre–randomization ventilation variables	4

eTable 3	Differences in MP values in the entire cohort	5

eTable 4	ROC curve results, threshold of MP 10 J/min, entire cohort	6

eTable 4b	ROC curve results, threshold of MP 10 J/min, only conventional	6

eTable 5	ROC curve results, threshold of MP 11 J/min, entire cohort	7

eTable 5b	ROC curve results, threshold of MP 11 J/min, only conventional	7

eTable 6	ROC curve results, threshold of MP 12 J/min, entire cohort	8

eTable 6b	ROC curve results, threshold of MP 12 J/min, only conventional	8

eTable 7	ROC curve results, threshold of MP 13 J/min, entire cohort	9

eTable 7b	ROC curve results, threshold of MP 13 J/min, only conventional	9

eTable 8	ROC curve results, threshold of MP 14 J/min, entire cohort	10

eTable 8b	ROC curve results, threshold of MP 14 J/min, only conventional	10

eTable 9	ROC curve results, threshold of MP 15 J/min, entire cohort	11

eTable 9b	ROC curve results, threshold of MP 15 J/min, only conventional	11

eTable 10	ROC curve results, threshold of MP 16 J/min, entire cohort	12

eTable 10b	ROC curve results, threshold of MP 16 J/min, only conventional	12

eTable 11	ROC curve results, threshold of MP 17 J/min, entire cohort	13

eTable 11b	ROC curve results, threshold of MP 17 J/min, only conventional	13

eTable 12	ROC curve results, threshold of MP 18 J/min, entire cohort	14

eTable 12b	ROC curve results, threshold of MP 18 J/min, only conventional	14

eTable 13	ROC curve results, threshold of MP 19 J/min, entire cohort	15

eTable 13b	ROC curve results, threshold of MP 19 J/min, only conventional	15

eTable 14	ROC curve results, threshold of MP 20 J/min, entire cohort	16

eTable 14b	ROC curve results, threshold of MP 20 J/min, only conventional	16

eTable 15	ROC curve results, threshold of MP 21 J/min, entire cohort	17

eTable 15b	ROC curve results, threshold of MP 21 J/min, only conventional	17

eTable 16	ROC curve results, threshold of MP 22 J/min, entire cohort	18

eTable 16b	ROC curve results, threshold of MP 22 J/min, only conventional	18

eTable 17	Results of Bland Altman analysis, only conventional ventilation	19

eTable 18	Correlation analysis results in the entire cohort.	20

eTable 18b	Correlation analysis results in only conventional ventilation	20

eFigure 1	Cumulative plots of all Mechanical Power equations	21

| **eTable 1.** Assumptions and purpose of proposed MP equations | | | | |  |  |
| --- | --- | --- | --- | --- | --- | --- |
| **Ref.** | **Equation** | **Ventilation mode** | **Complexity** | **Assumptions** | | **Purpose** |
| Gattinoni et al  (mathematically  simplified) | Eq. 1 | VCV | Simple | - Assumes linear Crs throughout inspiration  - Assumes constant Raw during inflation  - Assumes constant Flow  - Requires inspiratory/expiratory hold | | Simple bedside estimation of inspiratory MP |
| Becher et al (mathematically simplified) | Eq. 2 | PCV | Easy | - Assumes constant Raw through a squared pressure delivery  -Excludes inspiratory pressure rise time - No maneuver needed | | Simple bedside estimation of inspiratory MP |
| Guerin et al | Eq. 3 | Unspecified | Easy | - Requires inspiratory and expiratory hold | | Practical estimation using routinely available ventilator variables, as a surrogate of inspiratory intensity |
| Snoep et al | Eq. 4 | PCV | Easy to use | - Requires inspiratory hold | | Estimation of inspiratory MP using variables readily available at the bedside |
| Von During et al | Eq. 5 | Unspecified | Easy | - Approximated the conversion factor from 0.098 to 0.1  - Assumes linear Crs throughout inspiration  - Assumes constant Raw during inflation  - No maneuver needed | | Bedside estimation of inspiratory MP without requiring inspiratory hold maneuvers. |
| Giosa et al | Eq. 6 | VCV | Moderate | - Assumes linear Crs throughout inspiration  - Assumes constant Raw (10cmH2O·s/L)  - No maneuver needed | | Bedside estimation of inspiratory MP without the need for inspiratory hold manoeuvres |
| v/d Meijden et al | Eq. 7 | PCV | Complex | - Excludes inspiratory pressure rise time  - Includes nonlinear inspiratory limb of PV loop | | Incorporation of the inspiratory pressure–volume relationship by incorporating the nonlinear inspiratory limb of the PV loop, to estimate inspiratory MP |
| Gattinoni et al | Eq. 8 | VCV | Complex | - Assumes linear Crs throughout inspiration  - Assumes constant Raw during inflation  - Requires inspiratory/expiratory hold | | More complete estimation of inspiratory MP by incorporating additional respiratory mechanics variables |
| Trinkle et al | Eq. 9 | PCV | Complex | - Includes the pressure rise time and the path of the inspiratory limb | | Explicit correction for inspiratory pressure rise time to estimate inspiratory MP |
| Becher et al | Eq. 10 | PCV | Complex | - Accounts for both pressure rise time and the non-linear behavior of the inspiratory limb on the PV loop | | Most physiologically comprehensive estimation of inspiratory MP |

Abbreviations: Crs, Compliance of respiratory system; Eq, equation; MP, mechanical power; PCV, Pressure controlled ventilation; PV loop, Pressure/volume Loop; Ref, Reference; Raw, Airway resistance ; VCV, Volume controlled ventilation.

| **eTable 2.** Pre–randomization ventilation variables | | |
| --- | --- | --- |
|  | **N = 38** |  |
| *Ventilator settings and variables* | |  |
| MP, J/min | 18.6 [12.4–23.2] |  |
| V_T_, mL/kg PBW | 7.1 [5.7–8.2] |  |
| RR, bpm | 18 [14.2–22] |  |
| PEEP, cm H_2_O | 8 [6–10] |  |
| Pmax, cm H_2_O | 22 [17–26] |  |
| Pplat, cm H_2_O | 18 [15.4–22] |  |
| ΔP, cmH_2_O | 10 [9–11] |  |
| C_RS_, mL/cm H_2_O | 56.9 [40.3–74.3] |  |
| Arterial blood gas analyses  pH  PaCO_2_, kPa  Bicarbonate, mmol/L  PaO_2_, kPa | 7.4 [7.3–7.4]  5.3 [4.9–6.2  24.6 [21–27.6]  9.9 [9.1–11.6] |  |
| SaO_2,_ % | 95.3 [93.9–97] |  |
| FiO_2_, % | 35 [30–45] |  |
| PaO_2_ / FiO_2_ ratio | 212 [193–228] |  |

Data are expressed as median with [IQR] or numbers n/N (proportions) where appropriate.

Abbreviations: C_RS_, Compliance respiratory system; EtCO_2_, end-tidal carbon dioxide; FiO2, Fraction of inspired Oxygen; IQR, Interquartile range; MinVol, Minute Volume; MP Mechanical Power; PaCO_2_, Partial pressure of carbon dioxide; PaO_2_, Partial pressure of Oxygen; PBW, Predicted body weight; pH, potential of Hydrogen; PEEP, Positive End Expiratory Pressure; Pinsp, Inspiratory pressure; Pmax, Peak inspiratory pressure; Pplat, Plateau Pressure; Psupp, Pressure Support; RR, Respiratory Rate; SaO_2_, arterial Oxygen saturation; SpO_2_ Peripheral capillary oxygen saturation; V_Te_, Expiratory tidal volume; V_Ti_, Inspiratory tidal volume.

| **eTable 3.** Differences in MP values in the entire cohort. | | | |
| --- | --- | --- | --- |
|  | **MP** | **mean difference**  **(95% CI)** | **P value** |
| Geometric MP | 17.0 [12.3–21.5] | Ref | Ref |
| Eq. 1 | 15.8 [11.3–21.0] | -1.1 [-1.6 to -0.6] | <0.001 |
| Eq. 2 | 19.2 [14.4–25.4] | 2.5 [2.0 to 3.0] | <0.001 |
| Eq. 3 | 8.2 [6.1–10.8] | -8.8 [-9.4 to -8.3] | <0.001 |
| Eq. 4 | 16.5 [12.4–20.4] | -0.8 [-1.3 to -0.3] | 0.002 |
| Eq. 5 | 14.1 [10.2–18.2] | -3.2 [-3.7 to -2.6] | <0.001 |
| Eq. 6 | 18.1 [13.3–22.8] | 1.0 [0.5 to 1.5] | <0.001 |
| Eq. 7 | 18.3 [13.6–23.5] | 1.3 [0.7 to 1.8] | <0.001 |
| Eq. 8 | 15.6 [11.2–19.2] | -1.9 [-2.4 to -1.3] | <0.001 |
| Eq. 9 | 19.1 [14.3–25.2] | 2.1 [1.6 to 2.6] | <0.001 |
| Eq. 10 | 18.7 [14.1–24.7] | 2.0 [1.5 to 2.5] | <0.001 |

Data are expressed as median with [IQR]

Abbreviations: CI, confidence interval; Eq, equation; MP, mechanical power; Ref, reference.

| **eTable 4.** ROC curve results, threshold of MP 10 J/min, entire cohort | | | | |
| --- | --- | --- | --- | --- |
|  | **AUC** | **Specificity** | **Sensitivity** | **Youden** |
| Eq. 1 | 0.976 | 0.939 | 0.949 | 0.888 |
| Eq. 2 | 0.967 | 0.941 | 0.923 | 0.864 |
| Eq. 3 | 0.926 | 0.857 | 0.884 | 0.741 |
| Eq. 4 | 0.959 | 0.940 | 0.869 | 0.809 |
| Eq. 5 | 0.955 | 0.902 | 0.928 | 0.830 |
| Eq. 6 | 0.958 | 0.980 | 0.821 | 0.801 |
| Eq. 7 | 0.966 | 0.961 | 0.890 | 0.851 |
| Eq. 8 | 0.967 | 0.898 | 0.938 | 0.836 |
| Eq. 9 | 0.967 | 0.941 | 0.928 | 0.869 |
| Eq. 10 | 0.965 | 0.941 | 0.924 | 0.865 |

Data are expressed as numbers.

Abbreviations: AUC, Area Under the Curve.

| **eTable 4b.** ROC curve results, threshold of MP 10 J/min, only conventional | | | | |
| --- | --- | --- | --- | --- |
|  | **AUC** | **Specificity** | **Sensitivity** | **Youden** |
| Eq. 1 | 0.982 | 0.941 | 0.960 | 0.901 |
| Eq. 2 | 0.959 | 0.944 | 0.882 | 0.826 |
| Eq. 3 | 0.959 | 0.882 | 0.997 | 0.859 |
| Eq. 4 | 0.974 | 0.882 | 0.982 | 0.865 |
| Eq. 5 | 0.943 | 0.889 | 0.949 | 0.838 |
| Eq. 6 | 0.984 | 1.00 | 0.933 | 0.933 |
| Eq. 7 | 0.954 | 0.944 | 0.882 | 0.826 |
| Eq. 8 | 0.977 | 0.941 | 0.931 | 0.873 |
| Eq. 9 | 0.956 | 0.944 | 0.876 | 0.821 |
| Eq. 10 | 0.952 | 0.944 | 0.860 | 0.805 |

Data are expressed as numbers.

Abbreviations: AUC, Area Under the Curve.

| **eTable 5.** ROC curve results, threshold of MP 11 J/min, entire cohort | | | | |
| --- | --- | --- | --- | --- |
|  | **AUC** | **Specificity** | **Sensitivity** | **Youden** |
| Eq. 1 | 0.968 | 0.944 | 0.915 | 0.859 |
| Eq. 2 | 0.966 | 0.895 | 0.941 | 0.836 |
| Eq. 3 | 0.913 | 0.806 | 0.888 | 0.694 |
| Eq. 4 | 0.950 | 0.877 | 0.901 | 0.778 |
| Eq. 5 | 0.952 | 0.921 | 0.893 | 0.814 |
| Eq. 6 | 0.958 | 0.934 | 0.846 | 0.780 |
| Eq. 7 | 0.966 | 0.961 | 0.884 | 0.845 |
| Eq. 8 | 0.955 | 0.903 | 0.918 | 0.821 |
| Eq. 9 | 0.965 | 0.868 | 0.973 | 0.841 |
| Eq. 10 | 0.963 | 0.868 | 0.971 | 0.839 |

Data are expressed as numbers.

Abbreviations: AUC, Area Under the Curve.

| **eTable 5b.** ROC curve results, threshold of MP 11 J/min, only conventional | | | | |
| --- | --- | --- | --- | --- |
|  | **AUC** | **Specificity** | **Sensitivity** | **Youden** |
| Eq. 1 | 0.966 | 0.900 | 0.932 | 0.832 |
| Eq. 2 | 0.952 | 0.849 | 0.939 | 0.787 |
| Eq. 3 | 0.924 | 0.767 | 0.938 | 0.705 |
| Eq. 4 | 0.949 | 0.833 | 0.939 | 0.772 |
| Eq. 5 | 0.941 | 0.879 | 0.908 | 0.787 |
| Eq. 6 | 0.983 | 0.939 | 0.957 | 0.896 |
| Eq. 7 | 0.958 | 0.909 | 0.939 | 0.848 |
| Eq. 8 | 0.942 | 0.867 | 0.963 | 0.830 |
| Eq. 9 | 0.950 | 0.809 | 0.877 | 0.886 |
| Eq. 10 | 0.946 | 0.909 | 0.867 | 0.776 |

Data are expressed as numbers.

Abbreviations: AUC, Area Under the Curve.

| **eTable 6.** ROC curve results, threshold of MP 12 J/min, entire cohort | | | | |
| --- | --- | --- | --- | --- |
|  | **AUC** | **Specificity** | **Sensitivity** | **Youden** |
| Eq. 1 | 0.965 | 0.956 | 0.907 | 0.863 |
| Eq. 2 | 0.965 | 0.895 | 0.937 | 0.832 |
| Eq. 3 | 0.906 | 0.901 | 0.766 | 0.667 |
| Eq. 4 | 0.950 | 0.935 | 0.851 | 0.786 |
| Eq. 5 | 0.953 | 0.968 | 0.868 | 0.837 |
| Eq. 6 | 0.957 | 0.979 | 0.828 | 0.807 |
| Eq. 7 | 0.964 | 0.947 | 0.890 | 0.837 |
| Eq. 8 | 0.954 | 0.901 | 0.916 | 0.817 |
| Eq. 9 | 0.964 | 0.895 | 0.937 | 0.832 |
| Eq. 10 | 0.962 | 0.895 | 0.932 | 0.827 |

Data are expressed as numbers.

Abbreviations: AUC, Area Under the Curve.

| **eTable 6b.** ROC curve results, threshold of MP 12 J/min, only conventional | | | | |
| --- | --- | --- | --- | --- |
|  | **AUC** | **Specificity** | **Sensitivity** | **Youden** |
| Eq. 1 | 0.970 | 0.925 | 0.934 | 0.859 |
| Eq. 2 | 0.953 | 0.907 | 0.902 | 0.809 |
| Eq. 3 | 0.921 | 0.850 | 0.829 | 0.679 |
| Eq. 4 | 0.948 | 0.950 | 0.850 | 0.800 |
| Eq. 5 | 0.948 | 0.930 | 0.882 | 0.813 |
| Eq. 6 | 0.975 | 0.930 | 0.915 | 0.845 |
| Eq. 7 | 0.957 | 0.884 | 0.922 | 0.805 |
| Eq. 8 | 0.941 | 0.950 | 0.882 | 0.832 |
| Eq. 9 | 0.951 | 0.930 | 0.895 | 0.826 |
| Eq. 10 | 0.948 | 0.930 | 0.886 | 0.816 |

Data are expressed as numbers.

Abbreviations: AUC, Area Under the Curve.

| **eTable 7.** ROC curve results, threshold of MP 13 J/min, entire cohort | | | | |
| --- | --- | --- | --- | --- |
|  | **AUC** | **Specificity** | **Sensitivity** | **Youden** |
| Eq. 1 | 0.967 | 0.958 | 0.891 | 0.849 |
| Eq. 2 | 0.963 | 0.934 | 0.873 | 0.808 |
| Eq. 3 | 0.906 | 0.864 | 0.807 | 0.671 |
| Eq. 4 | 0.955 | 0.950 | 0.872 | 0.822 |
| Eq. 5 | 0.958 | 0.943 | 0.904 | 0.847 |
| Eq. 6 | 0.964 | 0.959 | 0.877 | 0.836 |
| Eq. 7 | 0.963 | 0.902 | 0.924 | 0.826 |
| Eq. 8 | 0.955 | 0.958 | 0.880 | 0.838 |
| Eq. 9 | 0.962 | 0.943 | 0.870 | 0.813 |
| Eq. 10 | 0.959 | 0.943 | 0.859 | 0.801 |

Data are expressed as numbers.

Abbreviations: AUC, Area Under the Curve.

| **eTable 7b.** ROC curve results, threshold of MP 13 J/min, only conventional | | | | |
| --- | --- | --- | --- | --- |
|  | **AUC** | **Specificity** | **Sensitivity** | **Youden** |
| Eq. 1 | 0.983 | 0.961 | 0.929 | 0.890 |
| Eq. 2 | 0.966 | 0.944 | 0.923 | 0.867 |
| Eq. 3 | 0.930 | 0.824 | 0.872 | 0.696 |
| Eq. 4 | 0.953 | 0.885 | 0.922 | 0.807 |
| Eq. 5 | 0.961 | 0.926 | 0.930 | 0.856 |
| Eq. 6 | 0.980 | 0.982 | 0.894 | 0.876 |
| Eq. 7 | 0.972 | 0.926 | 0.916 | 0.841 |
| Eq. 8 | 0.950 | 0.922 | 0.936 | 0.858 |
| Eq. 9 | 0.964 | 0.944 | 0.926 | 0.867 |
| Eq. 10 | 0.961 | 0.944 | 0.915 | 0.859 |

Data are expressed as numbers.

Abbreviations: AUC, Area Under the Curve.

| **eTable 8.** ROC curve results, threshold of MP 14 J/min, entire cohort | | | | |
| --- | --- | --- | --- | --- |
|  | **AUC** | **Specificity** | **Sensitivity** | **Youden** |
| Eq. 1 | 0.976 | 0.959 | 0.926 | 0.885 |
| Eq. 2 | 0.963 | 0.947 | 0.871 | 0.818 |
| Eq. 3 | 0.902 | 0.808 | 0.848 | 0.656 |
| Eq. 4 | 0.965 | 0.959 | 0.873 | 0.833 |
| Eq. 5 | 0.969 | 0.967 | 0.913 | 0.880 |
| Eq. 6 | 0.967 | 0.920 | 0.913 | 0.833 |
| Eq. 7 | 0.965 | 0.953 | 0.890 | 0.843 |
| Eq. 8 | 0.963 | 0.979 | 0.828 | 0.808 |
| Eq. 9 | 0.963 | 0.933 | 0.886 | 0.820 |
| Eq. 10 | 0.960 | 0.933 | 0.876 | 0.809 |

Data are expressed as numbers.

Abbreviations: AUC, Area Under the Curve.

| **eTable 8b.** ROC curve results, threshold of MP 14 J/min, only conventional | | | | |
| --- | --- | --- | --- | --- |
|  | **AUC** | **Specificity** | **Sensitivity** | **Youden** |
| Eq. 1 | 0.986 | 0.882 | 0.992 | 0.874 |
| Eq. 2 | 0.973 | 0.944 | 0.912 | 0.856 |
| Eq. 3 | 0.933 | 0.956 | 0.774 | 0.730 |
| Eq. 4 | 0.969 | 0.957 | 0.919 | 0.876 |
| Eq. 5 | 0.972 | 0.944 | 0.952 | 0.896 |
| Eq. 6 | 0.981 | 0.930 | 0.936 | 0.866 |
| Eq. 7 | 0.973 | 0.944 | 0.936 | 0.880 |
| Eq. 8 | 0.968 | 0.971 | 0.903 | 0.874 |
| Eq. 9 | 0.972 | 0.930 | 0.920 | 0.850 |
| Eq. 10 | 0.970 | 0.887 | 0.955 | 0.843 |

Data are expressed as numbers.

Abbreviations: AUC, Area Under the Curve.

| **eTable 9.** ROC curve results, threshold of MP 15 J/min, entire cohort | | | | |
| --- | --- | --- | --- | --- |
|  | **AUC** | **Specificity** | **Sensitivity** | **Youden** |
| Eq. 1 | 0.977 | 0.929 | 0.927 | 0.856 |
| Eq. 2 | 0.972 | 0.943 | 0.917 | 0.859 |
| Eq. 3 | 0.899 | 0.924 | 0.721 | 0.645 |
| Eq. 4 | 0.968 | 0.948 | 0.903 | 0.850 |
| Eq. 5 | 0.971 | 0.937 | 0.946 | 0.883 |
| Eq. 6 | 0.963 | 0.977 | 0.825 | 0.802 |
| Eq. 7 | 0.968 | 0.931 | 0.933 | 0.864 |
| Eq. 8 | 0.973 | 0.971 | 0.905 | 0.876 |
| Eq. 9 | 0.972 | 0.954 | 0.917 | 0.871 |
| Eq. 10 | 0.970 | 0.954 | 0.908 | 0.862 |

Data are expressed as numbers.

Abbreviations: AUC, Area Under the Curve.

| **eTable 9b.** ROC curve results, threshold of MP 15 J/min, only conventional | | | | |
| --- | --- | --- | --- | --- |
|  | **AUC** | **Specificity** | **Sensitivity** | **Youden** |
| Eq. 1 | 0.977 | 0.973 | 0.880 | 0.854 |
| Eq. 2 | 0.972 | 0.923 | 0.949 | 0.872 |
| Eq. 3 | 0.919 | 0.960 | 0.769 | 0.729 |
| Eq. 4 | 0.965 | 0.947 | 0.915 | 0.862 |
| Eq. 5 | 0.966 | 0.936 | 0.924 | 0.860 |
| Eq. 6 | 0.979 | 0.962 | 0.907 | 0.868 |
| Eq. 7 | 0.969 | 0.936 | 0.941 | 0.877 |
| Eq. 8 | 0.967 | 0.933 | 0.932 | 0.865 |
| Eq. 9 | 0.971 | 0.910 | 0.958 | 0.868 |
| Eq. 10 | 0.969 | 0.910 | 0.952 | 0.863 |

Data are expressed as numbers.

Abbreviations: AUC, Area Under the Curve.

| **eTable 10.** ROC curve results, threshold of MP 16 J/min, entire cohort | | | | |
| --- | --- | --- | --- | --- |
|  | **AUC** | **Specificity** | **Sensitivity** | **Youden** |
| Eq. 1 | 0.967 | 0.886 | 0.945 | 0.831 |
| Eq. 2 | 0.964 | 0.910 | 0.942 | 0.852 |
| Eq. 3 | 0.898 | 0.918 | 0.735 | 0.654 |
| Eq. 4 | 0.958 | 0.898 | 0.919 | 0.817 |
| Eq. 5 | 0.961 | 0.910 | 0.938 | 0.848 |
| Eq. 6 | 0.958 | 0.936 | 0.841 | 0.777 |
| Eq. 7 | 0.957 | 0.909 | 0.912 | 0.821 |
| Eq. 8 | 0.970 | 0.940 | 0.932 | 0.871 |
| Eq. 9 | 0.964 | 0.920 | 0.942 | 0.863 |
| Eq. 10 | 0.963 | 0.925 | 0.936 | 0.861 |

Data are expressed as numbers.

Abbreviations: AUC, Area Under the Curve.

| **eTable 10b.** ROC curve results, threshold of MP 16 J/min, only conventional | | | | |
| --- | --- | --- | --- | --- |
|  | **AUC** | **Specificity** | **Sensitivity** | **Youden** |
| Eq. 1 | 0.961 | 0.902 | 0.882 | 0.784 |
| Eq. 2 | 0.954 | 0.859 | 0.946 | 0.805 |
| Eq. 3 | 0.923 | 0.951 | 0.791 | 0.742 |
| Eq. 4 | 0.956 | 0.880 | 0.918 | 0.798 |
| Eq. 5 | 0.953 | 0.871 | 0.928 | 0.799 |
| Eq. 6 | 0.972 | 0.859 | 0.946 | 0.805 |
| Eq. 7 | 0.948 | 0.882 | 0.919 | 0.801 |
| Eq. 8 | 0.958 | 0.878 | 0.946 | 0.824 |
| Eq. 9 | 0.953 | 0.959 | 0.946 | 0.805 |
| Eq. 10 | 0.953 | 0.869 | 0.939 | 0.808 |

Data are expressed as numbers.

Abbreviations: AUC, Area Under the Curve.

| **eTable 11.** ROC curve results, threshold of MP 17 J/min, entire cohort | | | | |
| --- | --- | --- | --- | --- |
|  | **AUC** | **Specificity** | **Sensitivity** | **Youden** |
| Eq. 1 | 0.966 | 0.881 | 0.946 | 0.826 |
| Eq. 2 | 0.962 | 0.873 | 0.957 | 0.830 |
| Eq. 3 | 0.891 | 0.915 | 0.718 | 0.633 |
| Eq. 4 | 0.956 | 0.897 | 0.892 | 0.789 |
| Eq. 5 | 0.962 | 0.878 | 0.952 | 0.830 |
| Eq. 6 | 0.952 | 0.907 | 0.852 | 0.759 |
| Eq. 7 | 0.957 | 0.936 | 0.880 | 0.817 |
| Eq. 8 | 0.966 | 0.910 | 0.921 | 0.831 |
| Eq. 9 | 0.962 | 0.873 | 0.962 | 0.835 |
| Eq. 10 | 0.962 | 0.886 | 0.958 | 0.844 |

Data are expressed as numbers.

Abbreviations: AUC, Area Under the Curve.

| **eTable 11b.** ROC curve results, threshold of MP 17 J/min, only conventional | | | | |
| --- | --- | --- | --- | --- |
|  | **AUC** | **Specificity** | **Sensitivity** | **Youden** |
| Eq. 1 | 0.967 | 0.920 | 0.894 | 0.815 |
| Eq. 2 | 0.961 | 0.846 | 0.971 | 0.818 |
| Eq. 3 | 0.926 | 0.932 | 0.817 | 0.749 |
| Eq. 4 | 0.968 | 0.865 | 0.952 | 0.817 |
| Eq. 5 | 0.962 | 0.879 | 0.933 | 0.812 |
| Eq. 6 | 0.973 | 0.879 | 0.933 | 0.812 |
| Eq. 7 | 0.957 | 0.868 | 0.952 | 0.821 |
| Eq. 8 | 0.965 | 0.886 | 0.933 | 0.819 |
| Eq. 9 | 0.958 | 0.846 | 0.971 | 0.818 |
| Eq. 10 | 0.962 | 0.865 | 0.968 | 0.833 |

Data are expressed as numbers.

Abbreviations: AUC, Area Under the Curve.

| **eTable 12.** ROC curve results, threshold of MP 18 J/min, entire cohort | | | | |
| --- | --- | --- | --- | --- |
|  | **AUC** | **Specificity** | **Sensitivity** | **Youden** |
| Eq. 1 | 0.971 | 0.974 | 0.861 | 0.835 |
| Eq. 2 | 0.955 | 0.915 | 0.894 | 0.809 |
| Eq. 3 | 0.860 | 0.843 | 0.740 | 0.583 |
| Eq. 4 | 0.938 | 0.888 | 0.851 | 0.740 |
| Eq. 5 | 0.958 | 0.966 | 0.860 | 0.826 |
| Eq. 6 | 0.937 | 0.940 | 0.821 | 0.762 |
| Eq. 7 | 0.952 | 0.944 | 0.849 | 0.794 |
| Eq. 8 | 0.954 | 0.843 | 0.925 | 0.768 |
| Eq. 9 | 0.955 | 0.919 | 0.899 | 0.819 |
| Eq. 10 | 0.954 | 0.930 | 0.888 | 0.819 |

Data are expressed as numbers.

Abbreviations: AUC, Area Under the Curve.

| **eTable 12b.** ROC curve results, threshold of MP 18 J/min, only conventional | | | | |
| --- | --- | --- | --- | --- |
|  | **AUC** | **Specificity** | **Sensitivity** | **Youden** |
| Eq. 1 | 0.978 | 0.961 | 0.911 | 0.872 |
| Eq. 2 | 0.959 | 0.943 | 0.901 | 0.844 |
| Eq. 3 | 0.886 | 0.833 | 0.844 | 0.678 |
| Eq. 4 | 0.964 | 0.913 | 0.900 | 0.813 |
| Eq. 5 | 0.972 | 0.933 | 0.923 | 0.856 |
| Eq. 6 | 0.971 | 0.943 | 0.901 | 0.844 |
| Eq. 7 | 0.963 | 0.952 | 0.890 | 0.843 |
| Eq. 8 | 0.959 | 0.980 | 0.811 | 0.791 |
| Eq. 9 | 0.957 | 0.943 | 0.901 | 0.844 |
| Eq. 10 | 0.956 | 0.961 | 0.889 | 0.850 |

Data are expressed as numbers.

Abbreviations: AUC, Area Under the Curve.

| **eTable 13.** ROC curve results, threshold of MP 19 J/min, entire cohort | | | | |
| --- | --- | --- | --- | --- |
|  | **AUC** | **Specificity** | **Sensitivity** | **Youden** |
| Eq. 1 | 0.972 | 0.957 | 0.899 | 0.856 |
| Eq. 2 | 0.955 | 0.916 | 0.895 | 0.811 |
| Eq. 3 | 0.850 | 0.764 | 0.805 | 0.569 |
| Eq. 4 | 0.931 | 0.840 | 0.887 | 0.727 |
| Eq. 5 | 0.953 | 0.927 | 0.902 | 0.829 |
| Eq. 6 | 0.941 | 0.916 | 0.850 | 0.766 |
| Eq. 7 | 0.950 | 0.915 | 0.902 | 0.817 |
| Eq. 8 | 0.948 | 0.941 | 0.819 | 0.760 |
| Eq. 9 | 0.954 | 0.916 | 0.895 | 0.811 |
| Eq. 10 | 0.954 | 0.933 | 0.883 | 0.816 |

Data are expressed as numbers.

Abbreviations: AUC, Area Under the Curve.

| **eTable 13b.** ROC curve results, threshold of MP 19 J/min, only conventional | | | | |
| --- | --- | --- | --- | --- |
|  | **AUC** | **Specificity** | **Sensitivity** | **Youden** |
| Eq. 1 | 0.992 | 0.991 | 0.925 | 0.916 |
| Eq. 2 | 0.967 | 0.930 | 0.926 | 0.856 |
| Eq. 3 | 0.882 | 0.795 | 0.875 | 0.670 |
| Eq. 4 | 0.960 | 0.867 | 0.938 | 0.805 |
| Eq. 5 | 0.977 | 0.930 | 0.951 | 0.881 |
| Eq. 6 | 0.974 | 0.913 | 0.938 | 0.851 |
| Eq. 7 | 0.968 | 0.913 | 0.938 | 0.851 |
| Eq. 8 | 0.957 | 0.938 | 0.850 | 0.787 |
| Eq. 9 | 0.964 | 0.904 | 0.951 | 0.855 |
| Eq. 10 | 0.963 | 0.928 | 0.944 | 0.872 |

Data are expressed as numbers.

Abbreviations: AUC, Area Under the Curve.

| **eTable 14.** ROC curve results, threshold of MP 20 J/min, entire cohort | | | | |
| --- | --- | --- | --- | --- |
|  | **AUC** | **Specificity** | **Sensitivity** | **Youden** |
| Eq. 1 | 0.975 | 0.919 | 0.947 | 0.866 |
| Eq. 2 | 0.953 | 0.953 | 0.874 | 0.827 |
| Eq. 3 | 0.864 | 0.752 | 0.850 | 0.601 |
| Eq. 4 | 0.936 | 0.818 | 0.925 | 0.743 |
| Eq. 5 | 0.954 | 0.914 | 0.919 | 0.832 |
| Eq. 6 | 0.939 | 0.857 | 0.896 | 0.753 |
| Eq. 7 | 0.946 | 0.881 | 0.926 | 0.807 |
| Eq. 8 | 0.952 | 0.933 | 0.842 | 0.775 |
| Eq. 9 | 0.951 | 0.914 | 0.911 | 0.825 |
| Eq. 10 | 0.953 | 0.933 | 0.901 | 0.834 |

Data are expressed as numbers.

Abbreviations: AUC, Area Under the Curve.

| **eTable 14b.** ROC curve results, threshold of MP 20 J/min, only conventional | | | | |
| --- | --- | --- | --- | --- |
|  | **AUC** | **Specificity** | **Sensitivity** | **Youden** |
| Eq. 1 | 0.991 | 0.974 | 0.935 | 0.909 |
| Eq. 2 | 0.966 | 0.924 | 0.936 | 0.860 |
| Eq. 3 | 0.878 | 0.783 | 0.883 | 0.666 |
| Eq. 4 | 0.957 | 0.853 | 0.948 | 0.801 |
| Eq. 5 | 0.976 | 0.941 | 0.949 | 0.889 |
| Eq. 6 | 0.961 | 0.890 | 0.936 | 0.826 |
| Eq. 7 | 0.966 | 0.949 | 0.910 | 0.859 |
| Eq. 8 | 0.959 | 0.930 | 0.870 | 0.801 |
| Eq. 9 | 0.963 | 0.890 | 0.962 | 0.851 |
| Eq. 10 | 0.964 | 0.920 | 0.957 | 0.877 |

Data are expressed as numbers.

Abbreviations: AUC, Area Under the Curve.

| **eTable 15.** ROC curve results, threshold of MP 21 J/min, entire cohort | | | | |
| --- | --- | --- | --- | --- |
|  | **AUC** | **Specificity** | **Sensitivity** | **Youden** |
| Eq. 1 | 0.967 | 0.864 | 0.972 | 0.837 |
| Eq. 2 | 0.940 | 0.905 | 0.918 | 0.823 |
| Eq. 3 | 0.842 | 0.705 | 0.861 | 0.566 |
| Eq. 4 | 0.921 | 0.819 | 0.899 | 0.718 |
| Eq. 5 | 0.945 | 0.859 | 0.955 | 0.813 |
| Eq. 6 | 0.925 | 0.799 | 0.909 | 0.708 |
| Eq. 7 | 0.931 | 0.878 | 0.909 | 0.787 |
| Eq. 8 | 0.934 | 0.847 | 0.880 | 0.727 |
| Eq. 9 | 0.937 | 0.901 | 0.909 | 0.810 |
| Eq. 10 | 0.941 | 0.918 | 0.909 | 0.827 |

Data are expressed as numbers.

Abbreviations: AUC, Area Under the Curve.

| **eTable 15b.** ROC curve results, threshold of MP 21 J/min, only conventional | | | | |
| --- | --- | --- | --- | --- |
|  | **AUC** | **Specificity** | **Sensitivity** | **Youden** |
| Eq. 1 | 0.972 | 0.937 | 0.939 | 0.876 |
| Eq. 2 | 0.949 | 0.930 | 0.925 | 0.856 |
| Eq. 3 | 0.841 | 0.714 | 0.864 | 0.578 |
| Eq. 4 | 0.937 | 0.795 | 0.970 | 0.765 |
| Eq. 5 | 0.959 | 0.876 | 0.970 | 0.846 |
| Eq. 6 | 0.930 | 0.822 | 0.940 | 0.762 |
| Eq. 7 | 0.946 | 0.899 | 0.910 | 0.810 |
| Eq. 8 | 0.940 | 0.865 | 0.879 | 0.744 |
| Eq. 9 | 0.945 | 0.922 | 0.910 | 0.833 |
| Eq. 10 | 0.942 | 0.927 | 0.915 | 0.843 |

Data are expressed as numbers.

Abbreviations: AUC, Area Under the Curve.

| **eTable 16.** ROC curve results, threshold of MP 22 J/min, entire cohort | | | | |
| --- | --- | --- | --- | --- |
|  | **AUC** | **Specificity** | **Sensitivity** | **Youden** |
| Eq. 1 | 0.953 | 0.842 | 0.957 | 0.799 |
| Eq. 2 | 0.927 | 0.868 | 0.926 | 0.795 |
| Eq. 3 | 0.834 | 0.684 | 0.882 | 0.566 |
| Eq. 4 | 0.905 | 0.790 | 0.904 | 0.694 |
| Eq. 5 | 0.929 | 0.818 | 0.947 | 0.766 |
| Eq. 6 | 0.911 | 0.768 | 0.916 | 0.684 |
| Eq. 7 | 0.914 | 0.840 | 0.905 | 0.745 |
| Eq. 8 | 0.920 | 0.893 | 0.796 | 0.689 |
| Eq. 9 | 0.924 | 0.868 | 0.926 | 0.795 |
| Eq. 10 | 0.928 | 0.889 | 0.918 | 0.807 |

Data are expressed as numbers.

Abbreviations: AUC, Area Under the Curve.

| **eTable 16b.** ROC curve results, threshold of MP 22 J/min, only conventional | | | | |
| --- | --- | --- | --- | --- |
|  | **AUC** | **Specificity** | **Sensitivity** | **Youden** |
| Eq. 1 | 0.939 | 0.850 | 0.942 | 0.792 |
| Eq. 2 | 0.929 | 0.860 | 0.962 | 0.822 |
| Eq. 3 | 0.797 | 0.629 | 0.904 | 0.532 |
| Eq. 4 | 0.892 | 0.716 | 0.962 | 0.678 |
| Eq. 5 | 0.921 | 0.832 | 0.925 | 0.757 |
| Eq. 6 | 0.886 | 0.748 | 0.943 | 0.692 |
| Eq. 7 | 0.914 | 0.853 | 0.887 | 0.740 |
| Eq. 8 | 0.915 | 0.843 | 0.846 | 0.689 |
| Eq. 9 | 0.925 | 0.860 | 0.962 | 0.822 |
| Eq. 10 | 0.924 | 0.876 | 0.957 | 0.832 |

Data are expressed as numbers.

Abbreviations: AUC, Area Under the Curve.

| **eTable 17**. Results of Bland Altman analysis, only conventional ventilation | | | | | | | |
| --- | --- | --- | --- | --- | --- | --- | --- |
| **Equations** | **Mean of differences (bias)** | **Standard Deviation** | **Standard Error** | **Within Subject Variance** | **Between Subjects Variance** | **95% LOA (lower limit)** | **95% LOA (upper limit)** |
| Eq. 1 | 1.03 | 2.51 | 0.13 | 6.33 | 38.14 | -3.90 | 5.96 |
| Eq. 2 | -2.62 | 3.18 | 0.16 | 10.12 | 43.06 | -8.85 | 3.62 |
| Eq. 3 | 8.82 | 4.59 | 0.23 | 21.04 | 20.60 | -0.17 | 17.81 |
| Eq. 4 | 0.43 | 3.44 | 0.18 | 11.83 | 35.25 | -6.31 | 7.17 |
| Eq. 5 | 3.02 | 2.97 | 0.15 | 8.80 | 32.15 | -2.80 | 8.83 |
| Eq. 6 | -1.12 | 3.39 | 0.17 | 11.52 | 39.96 | -7.77 | 5.53 |
| Eq. 7 | -1.49 | 3.05 | 0.15 | 9.29 | 40.42 | -7.47 | 4.48 |
| Eq. 8 | 1.48 | 3.40 | 0.17 | 11.59 | 34.96 | -5.20 | 8.15 |
| Eq. 9 | -2.31 | 3.27 | 0.17 | 10.71 | 41.71 | -8.73 | 4.10 |
| Eq. 10 | -2.22 | 3.27 | 0.17 | 10.72 | 41.55 | -8.64 | 4.20 |

Data are expressed as numbers.

Abbreviations: Eq, Equation; LOA, Limits Of Agreement:

| **eTable 18.** Correlation analysis results in the entire cohort. | | | |
| --- | --- | --- | --- |
|  | **r** | **R²** | **P value** |
| GM vs Eq.1 | 0.907 | 0.823 | <0.001 |
| GM vs Eq.2 | 0.890 | 0.793 | <0.001 |
| GM vs Eq.3 | 0.711 | 0.505 | <0.001 |
| GM vs Eq.4 | 0.829 | 0.686 | <0.001 |
| GM vs Eq.5 | 0.876 | 0.768 | <0.001 |
| GM vs Eq.6 | 0.851 | 0.724 | <0.001 |
| GM vs Eq.7 | 0.879 | 0.773 | <0.001 |
| GM vs Eq.8 | 0.838 | 0.702 | <0.001 |
| GM vs Eq.9 | 0.883 | 0.780 | <0.001 |
| GM vs Eq.10 | 0.885 | 0.782 | <0.001 |

Data are expressed as numbers.

Abbreviations: GM, Geometric Method; Eq, Equation

| **eTable 18b.** Correlation analysis results in only conventional ventilation. | | | |
| --- | --- | --- | --- |
|  | **r** | **R²** | **P value** |
| GM vs Eq.1 | 0.920 | 0.847 | <0.001 |
| GM vs Eq.2 | 0.896 | 0.803 | <0.001 |
| GM vs Eq.3 | 0.697 | 0.486 | <0.001 |
| GM vs Eq.4 | 0.846 | 0.715 | <0.001 |
| GM vs Eq.5 | 0.884 | 0.781 | <0.001 |
| GM vs Eq.6 | 0.868 | 0.753 | <0.001 |
| GM vs Eq.7 | 0.893 | 0.798 | <0.001 |
| GM vs Eq.8 | 0.848 | 0.719 | <0.001 |
| GM vs Eq.9 | 0.884 | 0.781 | <0.001 |
| GM vs Eq.10 | 0.884 | 0.781 | <0.001 |

Data are expressed as numbers.

Abbreviations: GM, Geometric Method; Eq, Equation

**eFigure 1.** Cumulative plots of geometric MP and all equations

Vertical dotted line indicates the median of waveform equation; horizontal dotted line represents the proportion of 0.5.
Abbreviations: MP, mechanical power of ventilation.
